# Combining Miconazole and Domiphen Bromide Results in Excess of Reactive Oxygen Species and Killing of Biofilm Cells

**DOI:** 10.3389/fcell.2020.617214

**Published:** 2021-01-21

**Authors:** Jana Tits, Judith Berman, Bruno P. A. Cammue, Karin Thevissen

**Affiliations:** ^1^Centre of Microbial and Plant Genetics, Katholieke Universiteit Leuven, Leuven, Belgium; ^2^Department of Molecular Microbiology and Biotechnology, George S. Wise Faculty of Life Sciences, School of Molecular Cell Biology and Biotechnology, Tel Aviv University, Tel Aviv, Israel

**Keywords:** biofilm, *Candida*, combination treatment, fungicidal, miconazole, domiphen bromide, mode of action (MOA), reactive oxygen species

## Abstract

Fungal biofilm-related infections are increasingly occurring. We previously identified a fungicidal antibiofilm combination, consisting of miconazole (MCZ) and the quaternary ammonium compound domiphen bromide (DB). DB eliminates tolerance rather than altering the susceptibility to MCZ of various *Candida* spp. Here we studied the mode of action of the MCZ-DB combination in more detail. We found that DB's action increases the permeability of the plasma membrane as well as that of the vacuolar membrane of *Candida* spp. Furthermore, the addition of DB affects the intracellular azole distribution. MCZ is a fungicidal azole that, apart from its well-known inhibition of ergosterol biosynthesis, also induces accumulation of reactive oxygen species (ROS). Interestingly, the MCZ-DB combination induced significantly more ROS in *C. albicans* biofilms as compared to single compound treatment. Co-administration of the antioxidant ascorbic acid resulted in abolishment of the ROS generated by MCZ-DB combination as well as its fungicidal action. In conclusion, increased intracellular MCZ availability due to DB's action results in excess of ROS and enhanced fungal cell killing.

## Introduction

Various human body parts, like the oral cavity and reproductive organs, as well as implanted medical devices are potential substrates for fungal biofilm formation (Harriott et al., 2010; Rautemaa and Ramage, 2011; Ramage and Williams, 2013; Gökmanoglu et al., 2018). As the biofilm mode of growth provides protection against the host's immune defenses and fungal biofilm cells are phenotypically up to 1,000-fold more resistant to antifungal drugs compared to planktonic cultures, biofilm-related fungal infections are typically resistant to currently used antifungal treatments (Lamfon et al., 2004; Al-fattani and Douglas, 2006; Alhede et al., 2014; Johnson et al., 2016). Species from the genus *Candida*, of which *Candida albicans* is the most common, are a frequent cause of opportunistic biofilm-related infections, which affect an increasing amount of people due to the rising number of immunocompromised people and patients with implanted medical devices (Kojic and Darouiche, 2004; Cauda, 2009; Rautemaa and Ramage, 2011; Tumbarello et al., 2012; Lebeaux et al., 2014). Although fungal biofilms cause an increasing number of infections, only few new antifungal drugs with antibiofilm activity have been developed during the last decades (Ostrosky-zeichner et al., 2010; Butler and Cooper, 2011; Ramage and Williams, 2013; Roemer and Krysan, 2014; Kirchhoff et al., 2020). A valid alternative to the search for new types of antibiofilm drugs is the combination of a conventional antimycotic with a potentiator that increases the activity of the antimycotic against biofilms.

We previously identified the quaternary ammonium compound domiphen bromide (DB) as a miconazole (MCZ) potentiator (Tits et al., 2020). MCZ belongs to the azole type of antifungals, which is the preferred antifungal drug class for topical treatment of mucosal biofilm-related *Candida* infections (Gavarkar et al., 2013). Azoles inhibit the enzyme lanosterol 14-alpha-demethylase and most are fungistatic (Sud and Feingold, 1981; Hitchcock et al., 1990). However, the fungicidal imidazole MCZ additionally induces the accumulation of reactive oxygen species, resulting in killing of *Candida* cells (Kobayashi et al., 2002; François et al., 2006).

Fungicidal activity of MCZ against *Candida* biofilm cells has been observed only at very high MCZ concentrations (5 mM) (Vandenbosch et al., 2010), which are therapeutically unachievable, but can be used in antifungal lock therapy (Schinabeck et al., 2004; Buckler et al., 2008). Here, high antimicrobial drug concentrations are injected into the catheter lumen, in which microbial biofilms can develop (Schinabeck et al., 2004). We demonstrated fungicidal activity of the MCZ-DB combination at MCZ concentrations ranging from 62.5 to 500 μM (Tits et al., 2020). The MCZ-DB combination results in eradication of biofilms of *C. albicans* as well as of *C. glabrata* (Tits et al., 2020), an intrinsically azole-resistant *Candida* species (Ostrosky-Zeichner et al., 2003; Whaley and Rogers, 2016). Additionally, this combination is fungicidal against planktonic stationary cultures of *C. albicans* and *C. glabrata* (Tits et al., 2020). Moreover, MCZ-DB shows fungicidal activity against biofilms of fluconazole-resistant *C. albicans* isolates and of the emerging pathogen *C. auris* (Tits et al., 2020), which is inherently fluconazole-resistant and resistant to most antifungal drug classes (Lockhart et al., 2017; Ostrowsky et al., 2020). Furthermore, experimental evolution experiments indicate that resistance does not develop against this combination, pointing to the potential of this combination to be further developed as a potent antibiofilm treatment (Tits et al., 2020). As DB enhances the fungicidal activity of MCZ and other imidazoles, but not that of triazoles, against *C. albicans*, we posit that DB has an imidazole-specific potentiation activity with regard to fungal cell killing (Tits et al., 2020).

This study aims to unravel the mode of action of the MCZ-DB combination in more detail, using the most prevalent *Candida* species, *C. albicans* and *C. glabrata*, as a model. Here, DB's effect on azole uptake, intracellular azole distribution and accumulation of reactive oxygen species by MCZ was assessed.

## Materials and Methods

### Strains and Chemicals

*Candida albicans* strain SC5314 (Fonzi and Irwin, 1993) and *C. glabrata* strain BG2 (Kaur et al., 2007) were grown at 30°C on YPD [1% yeast extract, 2% bacteriological peptone (LabM, UK) and 2% glucose (Sigma-Aldrich, USA)] agar plates. Dimethyl sulfoxide (DMSO) (VWR International, Belgium) was used as a solvent to prepare stock solutions of miconazole (MCZ; Sigma-Aldrich), fluorescently labeled imidazole derivative (Benhamou et al., 2017), tetraethylammonium bromide (TCI Europe, Belgium), itraconazole (TCI Europe) and domiphen bromide (DB; Chemische Fabrik Berg GmbH). RPMI 1640 medium (pH 7.0) with L-glutamine and without sodium bicarbonate was purchased from Sigma-Aldrich and buffered with MOPS (Sigma-Aldrich). An ascorbic acid (Sigma-Aldrich) stock was made in Milli-Q water.

### Fungicidal Activity Assay

To determine whether a treatment is fungicidal against (i) biofilms or (ii) planktonic cultures in stationary phase of *C. albicans* SC5314 or *C. glabrata* BG2, a fungicidal activity assay was performed as described in Tits et al. (2020). (i) Briefly, *C. albicans* or *C. glabrata* overnight cultures were diluted to an optical density at 600 nm (OD_600nm_) of 0.1 in RPMI 1640 medium, followed by biofilm growth in a 100 μL volume at 37°C. Twenty-four hours old biofilms were treated with the appropriate single compound or combination (e.g., MCZ and DB). After an incubation period of 24 h (*C. albicans*) or 48 h (*C. glabrata*) at 37°C, the number of CFU was determined. To this end, biofilms were washed with phosphate-buffered saline (PBS) and scraped off from the bottom of the plate. Serial dilutions were plated on YPD agar plates, which were subsequently incubated for 24 h at 37°C. Finally, the number of colonies was determined. (ii) *C. albicans* or *C. glabrata* stationary-phase cultures at OD_600nm_ = 1 in RPMI 1640 medium were treated with the appropriate single compound or combination during 2.5 h shaking at 37°C. To determine the number of CFU, cultures were washed and resuspended in PBS, followed by plating of serial dilutions on YPD agar plates, which were subsequently incubated at 37°C. After an incubation period of 24 h, the number of colonies was determined.

### Flow Cytometry

To study the effect of DB on azole internalization in *C. glabrata* BG2 planktonic cultures, stationary cultures at OD_600nm_ = 1 in RPMI were treated with a 2-fold dilution series of a fluorescently labeled imidazole derivative (FKD) in presence or absence of 25 μM DB. 1 μM SYTOX Green nucleic acid stain (Thermo Fisher Scientific, USA) was added to the samples as a dead cell stain. After 2.5 h treatment shaking at 37°C, samples were washed with PBS and subjected to a flow cytometric analysis on a BD Influx™ cell sorter. 100,000 cells per samples were analyzed, measuring fluorescence at 530/40 nm (FL2_ λ_ex_ = 488 nm) and 670/30 nm (FL12_ λ_ex_ = 633 nm) to detect killing (SYTOX green +) and FKD internalization (FKD +), respectively.

### Confocal Microscopy

To investigate the subcellular localization of FKD, *C. glabrata* BG2 planktonic stationary cultures at OD_600nm_ = 1 in RPMI were treated with 250 μM FKD in the presence or absence of 25 μM DB, followed by an incubation period of 2.5 h shaking at 37°C. Samples were washed and resuspended in PBS and subsequently imaged with Confocal LSM880 airyscan, using ZEN black software. A 100x/1.46 oil immersion objective and a 2x or 8x computer zoom were used. We used the 633 nm laser to visualize FKD. Samples treated with FKD alone and with FKD in the presence of DB were analyzed with the same microscopy settings.

### Vacuolar pH Measurements

To assess the effects of DB on vacuolar pH, 2′,7′-Bis-(2-Carboxyethyl)-5-(and-6)-Carboxyfluorescein, Acetoxymethyl Ester (BCECF,AM; Thermo Fisher Scientific) staining was used as described previously (Struyfs et al., 2020). Concanamycin A (Sigma-Aldrich) was used as a positive control for an elevated vacuolar pH. *C. albicans* SC5314 and *C. glabrata* BG2 planktonic stationary cultures at OD_600nm_ = 1 in RPMI were treated with a 2-fold dilution series of DB. After 2 h treatment at 37°C, 50 μM BCECF,AM was added to each sample, followed by another 30′ incubation period at 37°C. Subsequently, cells were washed and resuspended in PBS. Of 100 μL cell suspension, optical density at 490 nm (OD490nm) and fluorescence intensity for excitation at 490 nm (I490nm) and 450 nm (I450nm), both measured at an emission wavelength of 535 nm, were measured. [(I490nm/I450nm)/OD490] was calculated and considered as a relative vacuolar pH value of the treated cells.

### Superoxide Detection

Superoxide detection was performed as described previously (De Cremer et al., 2016). Briefly, 24-h-old biofilms of *C. albicans* SC5314 or *C. glabrata* BG2 were treated with 150 or 500 μM MCZ, respectively, 37.5 μM DB or a combination of both in the presence or absence of 16 mM ascorbic acid (Sigma-Aldrich). After an incubation period of 24 h (*C. albicans*) or 48 h (*C. glabrata*) biofilms were washed and diluted in PBS. Then, biofilms were scraped off from the bottom of the microplate and 10 μL of this cell suspension was separated for CFU determination. To the remaining cells, a final concentration of 20 μM dihydroethidium (Thermo Fisher Scientific) was added, followed by an incubation period of 20 min at room temperature in the dark. Finally, cells were washed with PBS and fluorescence at λ_ex_ 510 nm and λ_em_ 595 nm was measured. Values were corrected for fluorescence detected in blank wells.

### Growth Inhibition Assay

To determine the effects of DB on the growth inhibitory potential of a triazole (e.g., itraconazole) against *C. albicans*, we performed a (i) planktonic growth inhibition assay and a (ii) biofilm inhibition assay. (i) The planktonic growth inhibition assay was performed according to the standard Clinical and Laboratory Standards Institute (CLSI) protocol M27-A3 (CLSI, 2008) as described in Vriens et al. (2015). (ii) A biofilm inhibition assay was performed as described in Cools et al. (2017) with some modifications. *C. albicans* overnight cultures were diluted to an OD_600nm_ = 0.1 in RPMI and added to the wells of a microplate, containing itraconazole, DB or a combination of both. After a 1-h adhesion phase (37°C), biofilm cells were washed with PBS and the same treatments were added. After 24 h of biofilm growth, biofilm formation was quantified with Cell-Titer Blue (CTB; Promega, USA).

### Data Analysis

To analyze the experimental data, GraphPad Prism (version 6) was used. Significant CFU differences were assessed based on the mean log values. Furthermore, significant CFU differences as well as differences in fluorescence units per 1,000 CFU upon dihydroethidium staining were determined using a two-way analysis of variance (ANOVA), followed by the appropriate multiple-comparison test. Significant differences in (I490nm/I450nm)/OD490nm were assessed by means of a one-way ANOVA and the appropriate multiple-comparison test. FlowJo™ 10 software and FIJI software (Schindelin et al., 2012) were used to analyze flow cytometry data and Airyscan images, respectively.

## Results

To unravel the mode of action of the MCZ-DB combination, we first focused on the effects of DB on membrane integrity and on azole internalization. To this end, we used a fluorescently labeled imidazole derivative, FKD (Probe 2 in Benhamou et al., 2017), which has properties reminiscent of ketoconazole and consists of a Np-Cy5 fluorescent dye attached to the azole core structure, i.e., a metadihalogenated phenyl ring and an azole ring (Supplementary Figure 1). FKD and ketoconazole have a similar antifungal activity profile and both affect cytochrome P450 and an additional target as shown by docking studies and susceptibility testing of cytochrome P450-lacking mutants (Benhamou et al., 2017). Moreover, Benhamou and coworkers reported FKD as a useful tool to investigate uptake, efflux and subcellular localization of imidazoles in *Candida* spp. (Benhamou et al., 2017). As imidazoles like ketoconazole can be potentiated by DB (Tits et al., 2020), we used FKD as a tool to study the mode of action of a DB-azole combination against *Candida* spp. However, we found that FKD can be potentiated by DB against planktonic stationary cultures of *C. glabrata* BG2, but, in contrast to miconazole, not against those of *C. albicans* SC5314 (Supplementary Figure 2). Therefore, we used planktonic *C. glabrata* BG2 cultures to further investigate the mode of action of the FKD-DB combination.

### DB Enables Internalization of a Fluorescently Labeled Imidazole Derivative in *C. glabrata* Cells

**Figure 1:**
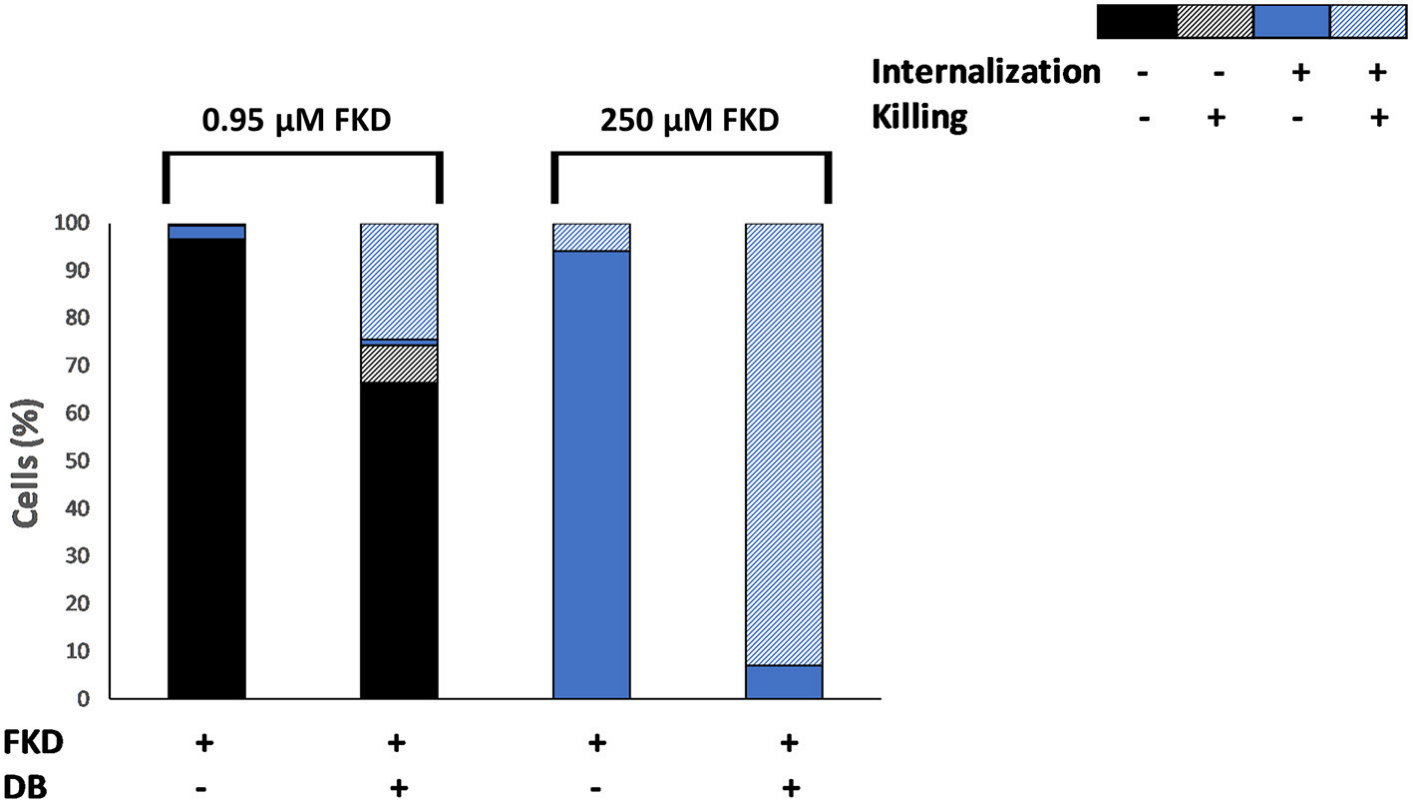
Effects of DB on internalization of fluorescently labeled imidazole derivative in planktonic stationary phase *C. glabrata* BG2 cultures. *C. glabrata* BG2 planktonic cultures were treated for 2.5 h with fluorescently labeled imidazole derivative (FKD) at 0.95 or 250 μM in the absence or presence of 25 μM DB. DMSO background concentration was 2%. Via FACS analysis, cell cultures were divided into four fractions according to FKD internalization and killing, as measured by SYTOX green. Results are a representative of three biological repeats.

The effects of DB on azole internalization and cell death in *C. glabrata* BG2 planktonic stationary cultures were studied via flow cytometry using two fluorescent dyes, FKD and SYTOX green, respectively. SYTOX green enters the cell through compromised membranes of dead cells, subsequently binds to DNA and fluoresces (Roth et al., 1997). A tight negative correlation (correlation coefficient r = −0.74) between the number of CFU and the number of SYTOX green fluorescent cells was observed upon FKD treatment in the presence of DB, indicating that SYTOX green can indeed be used to assess yeast cell death induced by uptake of FKD (Supplementary Figure 3). Cultures were treated with a series of FKD concentrations in presence or absence of a fixed DB concentration, with two conditions shown in Figure 1. At low FKD concentrations, significantly more cells had internalized FKD in the presence of 25 μM DB as compared to FKD treatment alone; while a minor fraction (22%) of the cell population was killed upon treatment with 25 μM DB alone, approximately all cells that internalized FKD were dead (SYTOX green positive) (Figure 1). This indicates that DB greatly augmented the cidality of MCZ, e.g., 0.95 μM MCZ and 25 μM DB (33% killed).

At high concentrations of FKD, it was internalized into all cells in the population, even in the absence of DB. However, while only 6% of the cell population was dead in the presence of FKD alone, 93% of the population was dead (SYTOX green positive) in the presence of both drugs (Figure 1). Furthermore, 7% of the cell population internalized FKD in the presence of DB, yet had no compromised membranes and was not dead (SYTOX green negative). In contrast to treatment with low FKD concentrations where increased FKD levels were observed inside the cells upon addition of DB, the FKD levels inside the *C. glabrata* cells treated with high FKD concentrations changed only minimally in the presence of DB compared to single FKD treatment (Supplementary Figure 4). However, the addition of DB significantly increased the number of dead cells at high FKD concentrations. This finding cannot be explained by increased FKD internalization alone and requires further investigation. How a small subpopulation (7%) survived the combination of FKD+DB also remains to be determined.

### DB Alters Cytoplasmic Distribution of Fluorescently Labeled Imidazole Derivative in *C. glabrata* Cells

**Figure 2:**
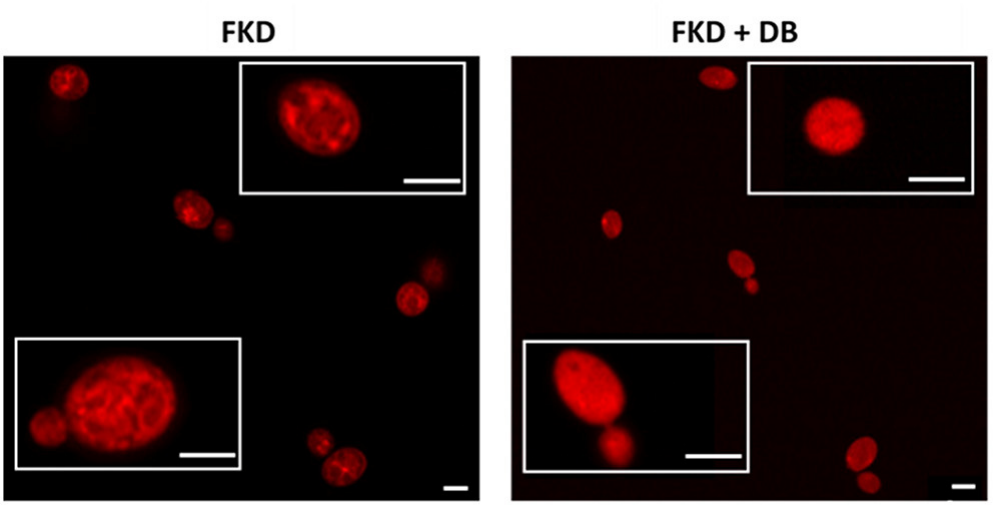
Effects of DB on cytoplasmic distribution of fluorescently labeled imidazole derivative in *C. glabrata* BG2 planktonic stationary phase cultures. Planktonic stationary cultures of *C. glabrata* BG2 were treated with 250 μM fluorescently labeled imidazole derivative (FKD) in the absence or presence of 25 μM domiphen bromide (DB). DMSO background concentration was 2%. Airyscan images of *C. glabrata* BG2 cells, both dividing (lower insets) and non-dividing (upper insets), treated for 2.5 h are shown. White bar: 2 μm.

Next we investigated the subcellular localization of FKD in presence and absence of DB, using confocal microscopy (Airyscan). Consistent with previous studies (Benhamou et al., 2017, 2018), FKD when treated alone localized to the mitochondria. By contrast, the combination of FKD and DB resulted in cells staining brightly and homogeneously throughout the cytoplasm (Figure 2), indicating that DB alters the cytoplasmic distribution of FKD in *C. glabrata* cells.

**Figure 3:**
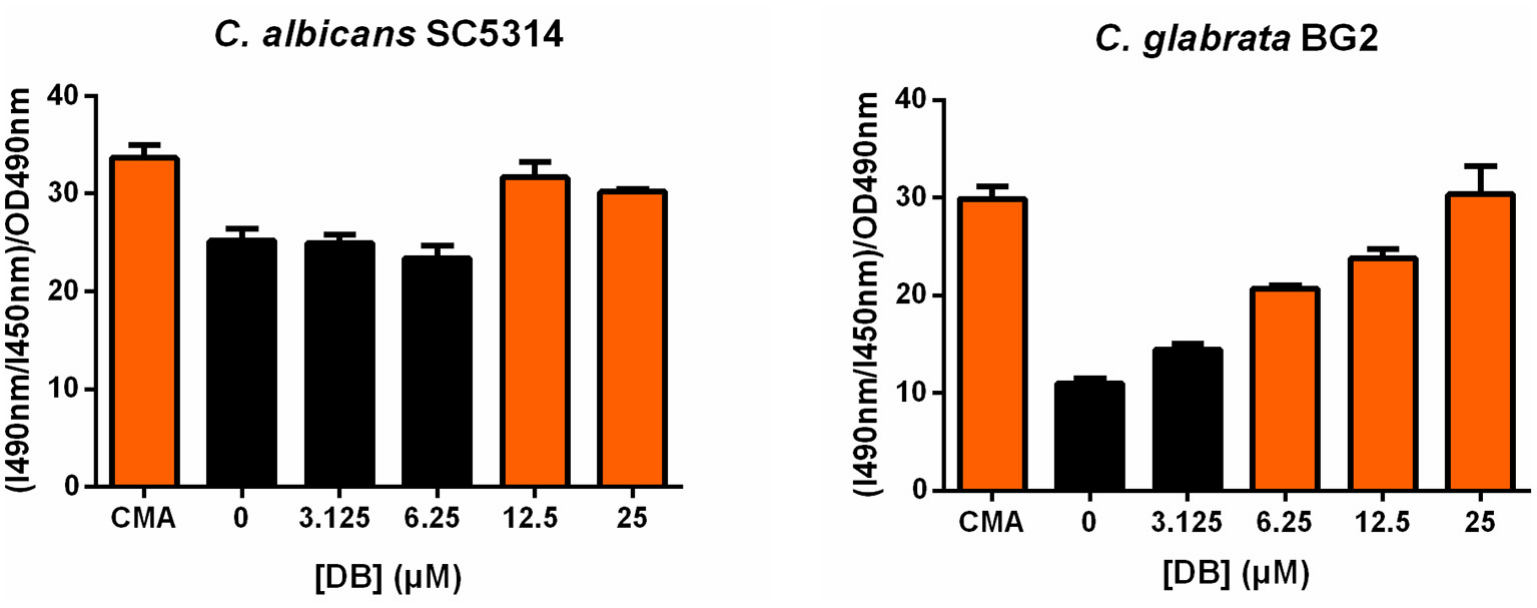
Effects of DB on vacuolar integrity in *C. albicans* SC5314 and *C. glabrata* BG2 planktonic cultures in stationary phase. Stationary cultures of *C. albicans* SC5314 and *C. glabrata* BG2 were treated with a 2-fold dilution series of domiphen bromide (DB) from 3.125 μM up to 25 μM for 2.5 h. DMSO background concentration was 2%. Concanamycin A (CMA) was used as a positive control for an elevated vacuolar pH. The ratio of fluorescence intensity of dye BCECF,AM from excitation at 490 nm (I490nm) to fluorescence intensity from excitation at 450 nm (I450nm), both measured at an emission wavelength of 535 nm, was calculated and normalized for the cell culture biomass (OD490nm) to obtain a relative vacuolar pH value of the treated cells. Mean values ± SEM are shown for three biological repeats. Statistical analysis was performed to assess a significant increase in (I490nm/I450nm)/OD490nm upon treatment with DB as compared to DMSO control treatment. A one-way ANOVA and Dunnett's multiple comparison test was applied and significant differences (*p* < 0.05) are shown in orange.

As vacuolar sequestration has been seen as an azole tolerance strategy in both *S. cerevisiae* and *C. albicans* (Khandelwal et al., 2019), we investigated whether DB itself affects vacuolar integrity using BCECF,AM, a fluorescent dye indicator specifically reporting vacuolar pH and hence, vacuolar function (Plant et al., 1999). More specifically, the ratio of fluorescence intensity of BCECF,AM from excitation at 490 nm (I490nm) to fluorescence intensity from excitation at 450 nm (I450nm), both measured at an emission wavelength of 535 nm, was calculated and normalized for the cell culture biomass (OD490nm). An increase in (I490nm/I450nm)/OD490nm indicates an elevated vacuolar pH and, accordingly, vacuolar dysfunction. Concanamycin A, a vacuolar H^+^-ATPase inhibitor, was used as a positive control for elevated vacuolar pH (Dröse and Altendorf, 1997). Upon treatment of both *C. albicans* SC5314 and *C. glabrata* BG2 cultures with sublethal DB concentrations, an increased (I490nm/I450nm)/OD490nm value was observed (Figure 3), suggesting that DB indeed negatively affects vacuolar function. Interestingly, quaternary ammonium compounds that cannot potentiate MCZ, such as tetraethylammonium bromide (Tits et al., 2020), did not affect vacuolar function (Supplementary Figure 5). These data support the hypothesis that DB enables release of vacuole-sequestered azoles, probably via its action on the vacuolar membrane. DB thereby increases intracellular availability of MCZ, resulting in cell killing as MCZ is characterized by fungicidal action (François et al., 2006). However, whether FKD in the absence of DB also localizes to the vacuoles of *C. glabrata* cells remains to be determined as well as the possible causal link between DB's negative effects on vacuolar function and the altered cytoplasmic FKD distribution.

Although FKD-DB was not characterized by increased fungicidal activity against stationary cultures of *C. albicans* as compared to treatment with FKD or DB alone, we also investigated FKD internalization and subcellular localization in this pathogen. In line with the absence of a potentiator action by DB in this setup, flow cytometry data indicated that FKD was internalized in approximately all *C. albicans* cells, irrespective of whether DB was present or not, but this did not result in cell killing: <1% of the cells was killed (data not shown). In addition, confocal microscopy data indicated that the addition of DB did not result in an altered cytoplasmic distribution of FKD in *C. albicans* cells as compared to single FKD treatment (data not shown). These data point to the inability of DB to affect FKD's intracellular distribution in *C. albicans*, which is linked to the absence of a potentiator action by DB in this setup.

### MCZ-DB Results in Increased Accumulation of Reactive Oxygen Species and Killing of *C. albicans* Biofilm Cells

**Figure 4:**
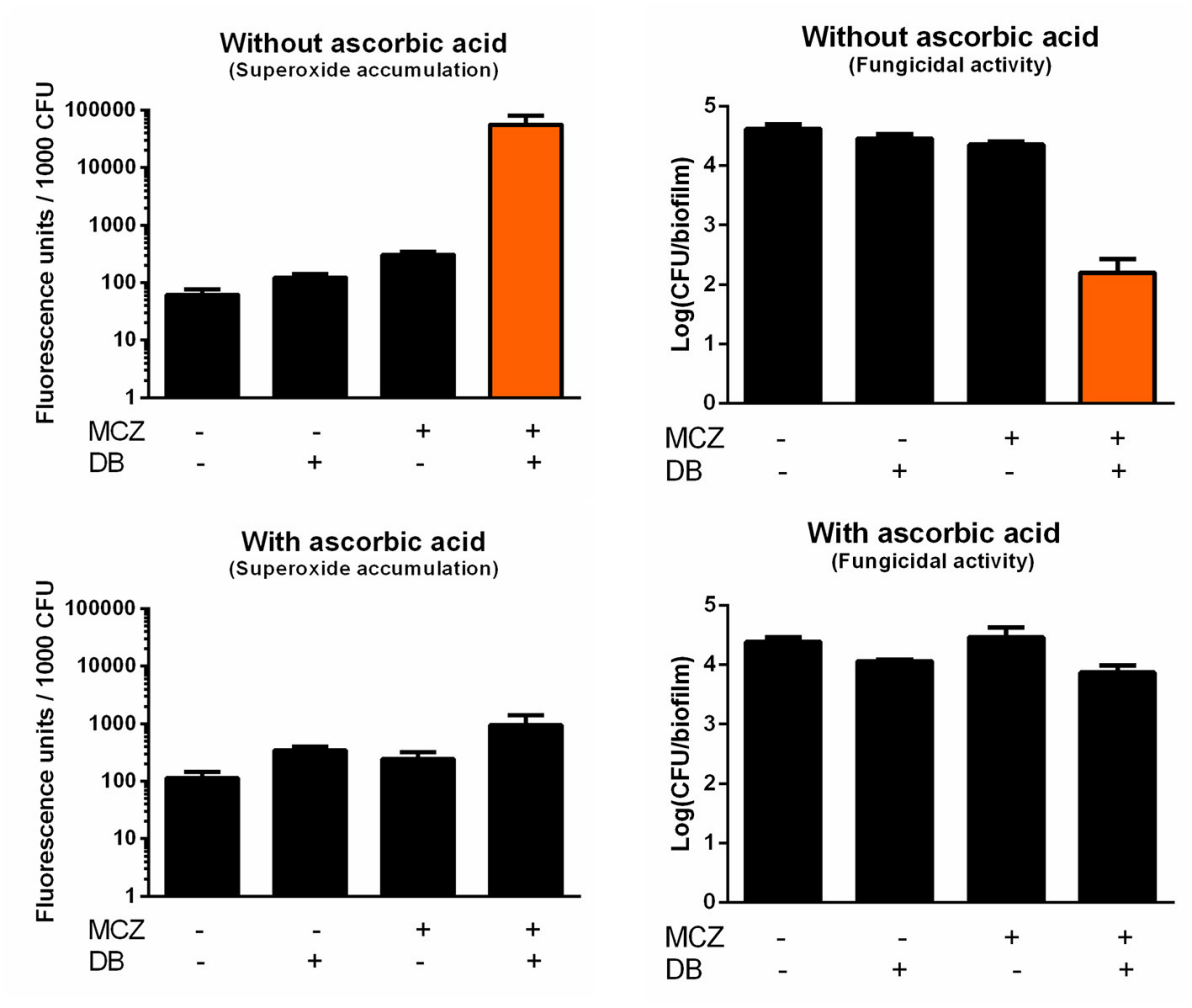
Superoxide accumulation in *C. albicans* SC5314 biofilm cells upon treatment with MCZ-DB and corresponding fungicidal activity in the absence and presence of ascorbic acid. *C. albicans* SC5314 biofilms were treated with 150 μM MCZ, 37.5 μM DB or a combination of both either in absence or presence of ascorbic acid. DMSO background concentration was 1%. Fluorescence at λ_ex_ 510 nm and λ_em_ 595 nm was normalized for the number of CFU/biofilm. Mean values ± SEM are shown for three biological repeats. Statistical analysis was performed to assess whether there is a significant increase in fluorescence units/1,000 CFU and a significant decrease in the number of CFU/biofilm upon treatment with MCZ-DB as compared to DMSO control treatment and both single compound treatments. A two-way ANOVA and Dunnett's or Tukey's multiple comparison test was applied and significant differences (*p* < 0.05) compared to single compound treatments and the DMSO control treatment are shown in orange.

**Figure 5:**
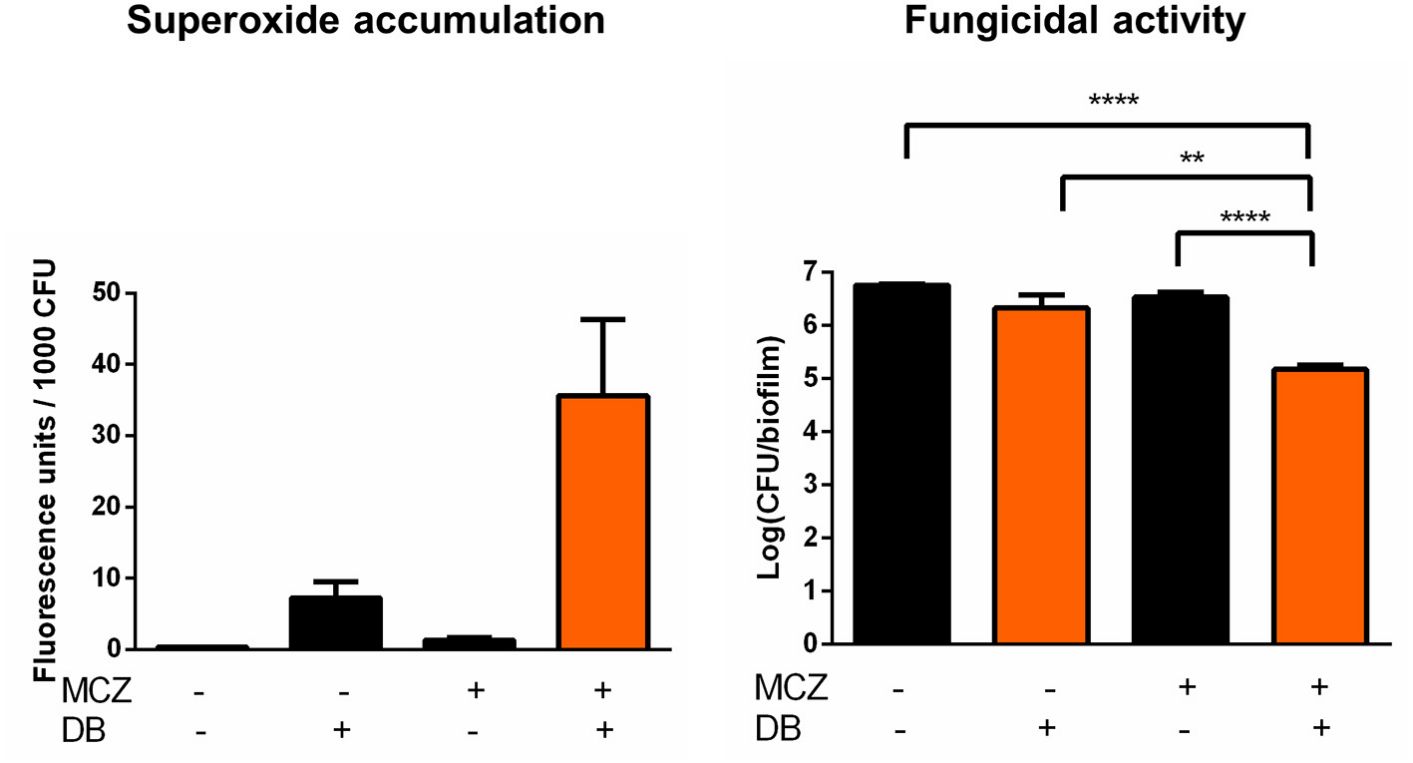
Superoxide accumulation in *C. glabrata* BG2 biofilm cells upon treatment with MCZ-DB and corresponding fungicidal activity. *C. glabrata* BG2 biofilms were treated with 500 μM MCZ, 37.5 μM DB or a combination of both. DMSO background concentration was 1%. Fluorescence at λ_ex_ 510 nm and λ_em_ 595 nm was normalized for the number of CFU/biofilm. Mean values ± SEM are shown for seven biological repeats. Statistical analysis was performed to assess whether there is a significant increase in fluorescence units/1,000 CFU and a significant decrease in the number of CFU/biofilm upon treatment with MCZ-DB as compared to DMSO control treatment and both single compound treatments. A two-way ANOVA and Dunnett's or Tukey's multiple comparison test was applied and significant differences (*p* < 0.01) compared to single compound treatments and the DMSO control treatment are shown in orange. **p < 0.01; ****p < 0.0001.

In contrast to most azoles, MCZ is characterized by fungicidal action against planktonic *Candida* cultures and, at very high supra-therapeutic concentrations (5 mM), against biofilms (François et al., 2006; Vandenbosch et al., 2010). In addition to its inhibition of ergosterol biosynthesis, MCZ induces the accumulation of reactive oxygen species (ROS) in planktonic fungal cultures (Kobayashi et al., 2002; François et al., 2006). Here, we investigated whether MCZ-DB treatment results in increased ROS, more specifically superoxide, accumulation in *C. albicans* biofilms, by staining treated cells with dihydroethidium (DHE). We previously revealed that MCZ induces the accumulation of superoxide in *C. albicans* biofilm cells and that this accumulation is essential for MCZ's fungicidal activity against *C. albicans* biofilms (De Cremer et al., 2016). Because of the dependence of DHE conversion on the number of living cells within a biofilm, fluorescence values were normalized for the amount of viable cells/biofilm as assessed by CFU plating (De Brucker et al., 2013; De Cremer et al., 2016; Tits et al., 2020). Treatment of *C. albicans* biofilms with MCZ-DB resulted in significantly increased superoxide accumulation as compared to both compounds alone and the DMSO control treatment (Figure 4). This increase in superoxide accumulation, relative to each compound alone, was also observed when treating *C. glabrata* biofilms with the combination (Figure 5). CFU determination of the treated biofilms indicated that the MCZ-DB combination was fungicidal against biofilms of *C. albicans* (Figure 4) as well as against those of *C. glabrata* (Figure 5), which is according to our previous data (Tits et al., 2020).

To determine whether the fungicidal activity of MCZ-DB resulted from the observed elevated superoxide levels upon combination treatment, *C. albicans* biofilms were treated with MCZ, DB or a combination in the presence of the antioxidant ascorbic acid, and stained with DHE. As shown in Figure 4, the presence of the antioxidant abolished the accumulation of superoxide upon treatment with the MCZ-DB combination (Figure 4), as well as its fungicidal activity. Hence, these data suggest a causal link between the accumulation of ROS in *C. albicans* biofilms treated with MCZ-DB and the combination's fungicidal activity against *C. albicans* biofilm cells.

## Discussion

Four main classes of antifungal drugs are currently used to treat fungal infections, specifically azoles (e.g., miconazole), polyenes (e.g., amphotericin B), echinocandins (e.g., caspofungin), and allylamines (e.g., terbinafine) (Chen and Sorrell, 2007). However, fungal biofilm-related infections are often recurrent and difficult to treat, as biofilms are tolerant to the majority of commonly used antifungal drugs (Muzny and Schwebke, 2015; Sherry et al., 2017). So far, liposomal amphotericin B formulations and echinocandins are the only effective treatment options for biofilm-related fungal infections (Kuhn et al., 2002; Fiori et al., 2011; Uppuluri et al., 2011). The azole miconazole (MCZ) is the preferred topical treatment against mucosal *Candida* infections, despite its moderate antibiofilm activity due to the range of tolerance pathways that protect biofilm cells against MCZ's action. For example, we previously observed increased expression of genes involved in ergosterol biosynthesis and drug efflux as well as decreased expression of several components of the electron transport chain upon MCZ treatment of *C. albicans* biofilms (De Cremer et al., 2016). Moreover, we observed a synergistic interaction between MCZ and electron transport chain inhibitors against biofilms, but not against planktonic or oxygen-deprived *C. albicans* cultures. Thus, we tested the hypothesis that MCZ tolerance is due to a biofilm-specific oxygen-dependent mechanism and found that electron transport chain inhibitors could enhance MCZ's antibiofilm activity in *C. albicans* (De Cremer et al., 2016).

Combining a common antifungal drug with a potentiator, a compound that enhances the drug's antibiofilm activity, is a novel strategy to extend the currently limited antibiofilm armamentarium. This approach may result in a lower risk for fungal resistance development, an extended antibiofilm activity spectrum, rapid antifungal action and lower toxic drug dosage (Bink et al., 2011). Recently, the discovery of novel potentiators of currently used antifungal drugs as well as deciphering promise of their mode of action has been the focus of several studies (Yu et al., 2013; Gu et al., 2016; Li et al., 2016; Tits et al., 2020). The antidepressant fluoxetine, for example, acts synergistically with azoles against planktonic cultures of azole-resistant *C. albicans* isolates and biofilms in early developmental stages by affecting the activity of secreted phospholipases and downregulating genes associated with secreted aspartyl proteinases, thereby reducing *C. albicans'* virulence (Gu et al., 2016) to decrease tolerance more dramatically than resistance (Rosenberg et al., 2018). Furthermore, a drug combination can be characterized by multiple modes of action. For instance, Yu and colleagues found that fluconazole and the antianginal drug verapamil synergized to inhibit *C. albicans* biofilm formation and preformed biofilms (Yu et al., 2013). This combination downregulates the gene encoding Agglutinin-like protein 3, which is important, e.g., for adherence to the host. Moreover, this fluconazole-verapamil treatment inhibits filamentation and biofilm formation as compared to single compound treatments of *C. albicans* (Yu et al., 2013). Hence, this combination affects various aspects contributing to *C. albicans'* virulence.

In this study, we aimed to unravel the precise mode of action of our recently identified fungicidal antibiofilm combination treatment, consisting of domiphen bromide (DB) and MCZ, against several *Candida* spp. (Tits et al., 2020). DB belongs to a group of cationic surfactants, called quaternary ammonium compounds, which are frequently used as surface disinfectants (Simoes et al., 2005; Gerba, 2015). Several studies suggest a negative impact of quaternary ammonium compounds on cell membranes (Hamilton, 1968; Ioannou et al., 2007; Ferreira et al., 2011). Indeed, at low doses, DB increases the internalization of a fluorescently labeled imidazole derivative (FKD). Other examples of antifungal combination treatments that affect import/efflux of an antifungal drug include retigeric acid B, which enhances the import of azoles, and budesonide, which inhibits fluconazole efflux out of fluconazole-resistant *C. albicans* cells (Sun et al., 2009; Li et al., 2016). Additionally, fluconazole-budesonide affects the activity of extracellular phospholipases and biofilm formation, thereby impairing *C. albicans* virulence (Li et al., 2016).

Vacuolar azole sequestration is a recently reported mechanism of azole tolerance that is conserved in pathogenic and non-pathogenic yeasts and results in a reduced effective azole concentration inside the cells (Luna-Tapia et al., 2015, 2016; Khandelwal et al., 2019). We demonstrated that DB's action increases vacuolar membrane permeability in planktonic stationary *C. glabrata* cultures, via its surfactant properties. Furthermore, an altered intracellular azole distribution was observed upon addition of DB. Whether there is a causal link between vacuolar permeabilization and the altered intracellular azole distribution remains to be determined. However, DB's effect on vacuolar permeability could potentially release vacuole-sequestered azoles. As FKD was previously shown to localize in mitochondria (Benhamou et al., 2017), DB might also release FKD from these organelles, resulting in a homogeneous FKD distribution throughout the cytoplasm. In the future, co-staining of *C. glabrata* cells with FKD and a vacuolar or mitochondrial dye will be required to assess whether FKD localizes to the mitochondria and/or the vacuoles in the absence of DB. In addition, such co-staining will shed light on the impact of DB on a potential release of FKD from these organelles. However, we can still conclude that, irrespective of the azole's precise localization, the addition of DB results in an altered subcellular azole localization.

**Figure 6:**
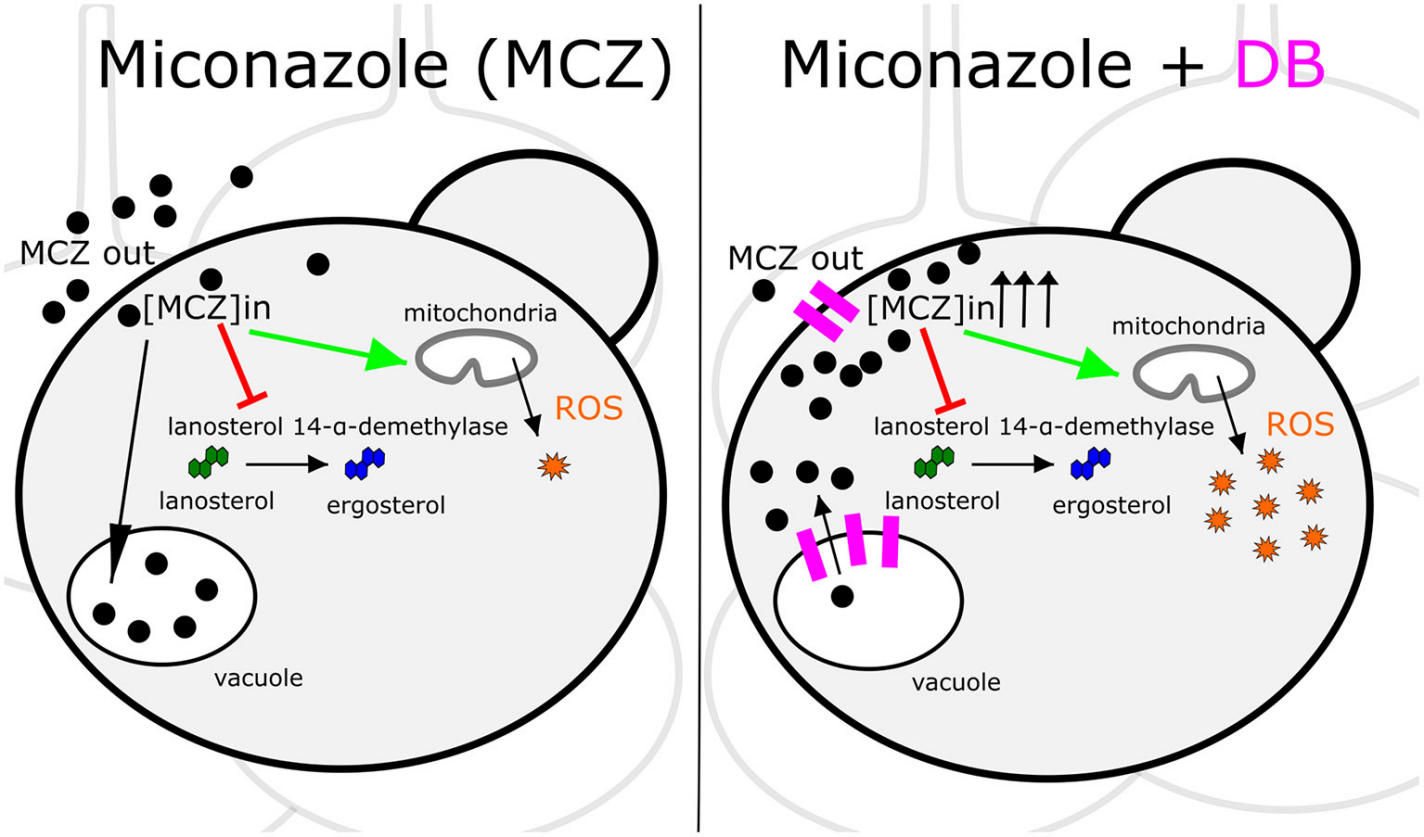
Schematic overview of the mode of action of MCZ alone juxtaposed with that of MCZ-DB. The mode of action of miconazole (MCZ) is illustrated on the left panel and that of MCZ in the presence of domiphen bromide (DB) is presented on the right. Each panel shows a *Candida* cell in a biofilm context. Red arrows and green arrows represent inhibition of ergosterol biosynthesis and induction of ROS accumulation, respectively. Pink indications on the right panel illustrate DB's effects on the cellular membranes. Sequestration of MCZ into and release of MCZ from the vacuole is illustrated by a black arrow pointing toward or away from the vacuole, respectively, although further investigation is required to confirm that DB treatment results in the release of vacuole-sequestered MCZ. ROS, reactive oxygen species; [MCZ]in, intracellular miconazole concentration.

MCZ is a fungicidal azole that, apart from inhibiting ergosterol biosynthesis, induces the accumulation of reactive oxygen species (ROS) (François et al., 2006). Hence, increased intracellular MCZ availability is expected to result in enhanced cell killing. Indeed, we discovered that the fungicidal MCZ-DB combination results in increased ROS accumulation relative to either drug alone in both *C. albicans* and *C. glabrata* biofilms. The disappearance of the fungicidal effect of MCZ-DB, as well as the elevated ROS levels, in the presence of the antioxidant ascorbic acid, points to a causal link between increased ROS accumulation and the fungicidal activity of MCZ-DB. Figure 6 presents a schematic overview of the mode of action of MCZ alone, juxtaposed with that of MCZ-DB.

It is very important to note that our data on an imidazole-specific potentiation activity of DB (Tits et al., 2020) are based on CFU readouts in a biofilm setup, implying fungicidal activity of the combination against mature biofilm cells. This is in line with the reported fungicidal activity of miconazole. Increasing the intracellular concentration of fungistatic triazoles via DB however will never result in killing of biofilm cells (Tits et al., 2020). Nevertheless, we found that DB can increase the growth inhibitory activity of the triazole itraconazole against *C. albicans* in a planktonic and biofilm culture growth inhibitory setup, resulting in a more than 4-fold decreased MIC of itraconazole (from 0.78 μM toward 0.17 μM in the presence of 3.1 μM DB) and increased biofilm inhibitory activity (Supplementary Figure 6). Hence, it is likely that DB also releases triazoles from organelles like the vacuole, resulting in increased cytoplasmic azole concentrations in general, and in increased fungistatic (triazoles) or fungicidal (imidazoles) activity in particular.

In conclusion, our mode of action data support the hypothesis that DB increases intracellular MCZ availability by enhancing azole import and possibly by releasing sequestered MCZ, resulting in elevated ROS levels and subsequent killing of the fungal biofilm cell.

## Data Availability Statement

The original contributions presented in the study are included in the article/Supplementary Materials, further inquiries can be directed to the corresponding author.

## Author Contributions

KT and BC supervised the study. JT performed the experiments and analysis. JT wrote the manuscript. KT and JB finalized the manuscript. All authors were involved in designing the study, read, and approved the final manuscript.

## Conflict of Interest

The authors declare that the research was conducted in the absence of any commercial or financial relationships that could be construed as a potential conflict of interest.

## References

[B1] Al-fattani M. A. Douglas L. J. (2006). Biofilm matrix of *Candida albicans* and *Candida tropicalis*: chemical composition and role in drug resistance. J. Med. Microbiol. 55, 999–1008. 10.1099/jmm.0.46569-0 16849719

[B2] Alhede M. Bjarnsholt T. Givskov M. Alhede M. (2014). *Pseudomonas aeruginosa* biofilms: mechanisms of immune evasion. Adv. Appl. Microbiol. 86, 1–40. 10.1016/B978-0-12-800262-9.00001-9 24377853

[B3] Benhamou R. I. Bibi M. Berman J. Fridman M. (2018). Localizing antifungal drugs to the correct organelle can markedly enhance their efficacy. Angew. Chem. Int. Ed. Engl. 57, 6230–6235. 10.1002/anie.201802509 29575397 PMC7035955

[B4] Benhamou R. I. Bibi M. Steinbuch K. B. Engel H. Levin M. Roichman Y.. (2017). Real-time imaging of the azole class of antifungal drugs in live candida cells. ACS Chem. Biol. 12, 1769–1777. 10.1021/acschembio.7b00339 28472585 PMC7030953

[B5] Bink A. Pellens K. Cammue B. P. A. Thevissen K. (2011). Anti-biofilm strategies: how to eradicate candida biofilms? Open Mycol. J. 5, 29–38. 10.2174/1874437001105010029

[B6] Buckler B. S. Sams R. N. Goei V. L. Krishnan K. R. Bemis M. J. Parker D. P.. (2008). Treatment of central venous catheter fungal infection using liposomal amphotericin-B lock therapy. Pediatr. Infect. Dis. J. 27, 762–764. 10.1097/INF.0b013e318170b68b 18664989

[B7] Butler M. S. Cooper M. A. (2011). Antibiotics in the clinical pipeline in 2011. J. Antibiot. 64, 413–425. 10.1038/ja.2011.44 21587262

[B8] Cauda R. (2009). Candidaemia in patients with an inserted medical device. Drugs 69(Suppl. 1), S33–38. 10.2165/11315520-000000000-00000 19877732

[B9] Chen S. C. A. Sorrell T. C. (2007). Antifungal agents. Med. J. Aust. 187, 404–409. 10.5694/j.1326-5377.2007.tb01313.x 17908006

[B10] CLSI (2008). Reference Method for Broth Dilution Antifungal Susceptibility Testing of Yeasts; Approved Standard. Wayne, PA: Clinical and Laboratory Standard Institute.

[B11] Cools T. L. Struyfs C. Drijfhout J. W. Kucharíková S. Romero C. L. Van Dijck P.. (2017). A linear 19-mer plant defensin-derived peptide acts synergistically with caspofungin against *Candida albicans* biofilms. Front. Microbiol. 8:2051. 10.3389/fmicb.2017.02051 29104569 PMC5655031

[B12] De Brucker K. Bink A. Meert E. Cammue B. P. A. Thevissen K. (2013). Potentiation of antibiofilm activity of amphotericin B by superoxide dismutase inhibition. Oxid. Med. Cell. Longev. 2013:704654. 10.1155/2013/704654 24078861 PMC3774027

[B13] De Cremer K. De Brucker K. Staes I. Peeters A. Van Den Driessche F. Coenye T.. (2016). Stimulation of superoxide production increases fungicidal action of miconazole against *Candida albicans* biofilms. Sci. Rep. 6:27463. 10.1038/srep27463 27272719 PMC4895440

[B14] Dröse S. Altendorf K. (1997). Bafilomycins and concanamycins as inhibitors of V-ATPases and P-ATPases. J Exp Biol 200, 1–8. 9023991 10.1242/jeb.200.1.1

[B15] Ferreira C. Pereira A. M. Pereira M. C. Melo L. F. Simoes M. (2011). Physiological changes induced by the quaternary ammonium compound benzyldimethyldodecylammonium chloride on Pseudomonas fluorescens. J. Antimicrob. Chemother. 66, 1036–1043. 10.1093/jac/dkr028 21393196

[B16] Fiori B. Posteraro B. Torelli R. Tumbarello M. Perlin D. S. Fadda G.. (2011). *In vitro* activities of anidulafungin and other antifungal agents against biofilms formed by clinical isolates of different *Candida* and *Aspergillus* species. Antimicrob. Agents Chemother. 55, 3031–3035. 10.1128/AAC.01569-10 21422210 PMC3101406

[B17] Fonzi W. A. Irwin M. Y. (1993). Isogenic strain construction and gene mapping in *Candida albicans*. Genetics 134, 717–728. 8349105 10.1093/genetics/134.3.717 PMC1205510

[B18] François I. E. J. A. Cammue B. P. A. Borgers M. Ausma J. Dispersyn G. D. Thevissen K. (2006). Azoles: mode of antifungal action and resistance development. Effect of miconazole on endogenous reactive oxygen species production in *Candida albicans*. Anti Infect Agents Med. Chem. 5, 3–13. 10.2174/187152106774755554

[B19] Gavarkar P. S. Adnaik R. S. Mohite S. K. (2013). An overview of azole antifungals. Int. J. Pharm. Sci. Res. 4, 4083–4089. 10.13040/IJPSR.0975-8232.4(11).4083-89

[B20] Gerba C. P. (2015). Quaternary ammonium biocides: efficacy in application. Appl. Environ. Microbiol. 81, 464–469. 10.1128/AEM.02633-14 25362069 PMC4277564

[B21] Gökmanoglu C. Kara N. Beldüz M. Kamburoglu A. Tosun I. Sadik E.. (2018). Evaluation of *Candida albicans* biofilm formation on various parts of implant material surfaces. Niger. J. Clin. Pract. 21, 33–37. 10.4103/1119-3077.224793 29411720

[B22] Gu W. Guo D. Zhang L. Xu D. Sun S. (2016). The synergistic effect of azoles and fluoxetine against resistant Candida albicans strains is attributed to attenuating fungal virulence. Antimicrob. Agents Chemother. 60, 6179–6188. 10.1128/AAC.03046-15 27503639 PMC5038273

[B23] Hamilton W. A. (1968). The mechanism of the bacteriostatic action of tetrachlorosalicylanilide: a membrane-active antibacterial compound. J. Gen. Microbiol. 50, 441–458. 10.1099/00221287-50-3-441 4870833

[B24] Harriott M. M. Lilly E. A. Rodriguez T. E. Fidel P. L. Noverr M. C. (2010). *Candida albicans* forms biofilms on the vaginal mucosa. Microbiology 156, 3635–3644. 10.1099/mic.0.039354-0 20705667 PMC3068702

[B25] Hitchcock C. A. Dickinson K. Brown S. B. Evans E. G. V. Adams D. J. (1990). Interaction of azole antifungal antibiotics with cytochrome P-450- dependent 14a-sterol demethylase purified from *Candida albicans*. Biochem. J. 266, 475–480. 10.1042/bj2660475 2180400 PMC1131156

[B26] Ioannou C. J. Hanlon G. W. Denyer S. P. (2007). Action of disinfectant quaternary ammonium compounds against *Staphylococcus aureus*. Antimicrob. Agents Chemother. 51, 296–306. 10.1128/AAC.00375-06 17060529 PMC1797692

[B27] Johnson C. J. Cabezas-Olcoz J. Kernien J. F. Wang S. X. Beebe D. J. Huttenlocher A.. (2016). The extracellular matrix of *Candida albicans* biofilms impairs formation of neutrophil extracellular traps. PLoS Pathog. 12:e1005884. 10.1371/journal.ppat.1005884 27622514 PMC5021349

[B28] Kaur R. Ma B. Cormack B. P. (2007). A family of glycosylphosphatidylinositol-linked aspartyl proteases is required for virulence of *Candida glabrata*. Proc. Natl. Acad. Sci. U.S.A. 104, 7628–7633. 10.1073/pnas.0611195104 17456602 PMC1863504

[B29] Khandelwal N. K. Wasi M. Nair R. Gupta M. Kumar M. Mondal A. K.. (2019). Vacuolar sequestration of azoles, a novel strategy of azole antifungal resistance conserved across pathogenic and nonpathogenic yeast. Antimicrob. Agents Chemother. 63, e01347–e01318. 10.1128/AAC.01347-18 30642932 PMC6395933

[B30] Kirchhoff L. Dittmer S. Weisner A. K. Buer J. Rath P. M. Steinmann J. (2020). Antibiofilm activity of antifungal drugs, including the novel drug olorofim, against Lomentospora prolificans. J. Antimicrob. Chemother. 75, 2133–2140. 10.1093/jac/dkaa157 32386411

[B31] Kobayashi D. Kondo K. Uehara N. Otokozawa S. Tsuji N. Yagihashi A.. (2002). Endogenous reactive oxygen species is an important mediator of miconazole antifungal effect. Antimicrob. Agents Chemother. 46, 3113–3117. 10.1128/AAC.46.10.3113-3117.2002 12234832 PMC128784

[B32] Kojic E. M. Darouiche R. O. (2004). Candida infections of medical devices. Clin. Microbiol. Rev. 17, 255–267. 10.1128/CMR.17.2.255-267.2004 15084500 PMC387407

[B33] Kuhn D. M. George T. Chandra J. Mukherjee P. K. Ghannoum M. A. (2002). Antifungal susceptibility of candida biofilms: unique efficacy of amphotericin B lipid formulations and echinocandins. Antimicrob. Agents Chemother. 46, 1773–1780. 10.1128/AAC.46.6.1773-1780.2002 12019089 PMC127206

[B34] Lamfon H. Porter S. R. Mccullough M. Pratten J. (2004). Susceptibility of *Candida albicans* biofilms grown in a constant depth film fermentor to chlorhexidine, fluconazole and miconazole: a longitudinal study. J. Antimicrob. Chemother. 53, 383–385. 10.1093/jac/dkh071 14729749

[B35] Lebeaux D. Fernández-hidalgo N. Chauhan A. Lee S. Ghigo J. Almirante B.. (2014). Management of infections related to totally implantable venous-access ports: challenges and perspectives. Lancet Infect. Dis. 14, 146–159. 10.1016/S1473-3099(13)70266-4 24314751

[B36] Li X. Yu C. Huang X. Sun S. (2016). Synergistic effects and mechanisms of budesonide in combination with fluconazole against resistant candida albicans. PLoS ONE 11:e0168936. 10.1371/journal.pone.0168936 28006028 PMC5179115

[B37] Lockhart S. R. Etienne K. A. Vallabhaneni S. Farooqi J. Chowdhary A. Govender N. P.. (2017). Simultaneous emergence of multidrug-resistant *Candida auris* on 3 continents confirmed by whole-genome sequencing and epidemiological analyses. Clin. Infect. Dis. 64, 134–140. 10.1093/cid/ciw691 27988485 PMC5215215

[B38] Luna-Tapia A. Kerns M. E. Eberle K. E. Jursic B. S. Palmer G. E. (2015). Trafficking through the late endosome significantly impacts Candida albicans tolerance of the azole antifungals. Antimicrob. Agents Chemother. 59, 2410–2420. 10.1128/AAC.04239-14 25666149 PMC4356793

[B39] Luna-Tapia A. Tournu H. Peters T. L. Palmer G. E. (2016). Endosomal trafficking defects can induce calcium-dependent azole tolerance in *Candida albicans*. Antimicrob. Agents Chemother. 60, 7170–7177. 10.1128/AAC.01034-16 27645241 PMC5118996

[B40] Muzny C. A. Schwebke J. R. (2015). Biofilms: an underappreciated mechanism of treatment failure and recurrence in vaginal infections. Clin. Infect. Dis. 61, 601–606. 10.1093/cid/civ353 25935553 PMC4607736

[B41] Ostrosky-zeichner L. Casadevall A. Galgiani J. N. Odds F. C. Rex J. H. (2010). An insight into the antifungal pipeline: selected new molecules and beyond. Nat. Rev. Drug Discov. 9, 719–727. 10.1038/nrd3074 20725094

[B42] Ostrosky-Zeichner L. Rex J. H. Pappas P. G. Hamill R. J. Larsen R. A. Horowitz H. W.. (2003). Antifungal susceptibility survey of 2,000 bloodstream Candida isolates in the United States. Antimicrob. Agents Chemother. 47, 3149–3154. 10.1128/AAC.47.10.3149-3154.2003 14506023 PMC201160

[B43] Ostrowsky B. Greenko J. Adams E. Quinn M. O'Brien B. Chaturvedi V.. (2020). *Candida auris* isolates resistant to three classes of antifungal medications — New York, 2019. MMWR Morb. Mortal Wkly. Rep. 69, 6–9. 10.15585/mmwr.mm6901a2 31917780 PMC6973342

[B44] Plant P. J. Manolson M. F. Grinstein S. Demaurex N. (1999). Alternative mechanisms of vacuolar acidification in H + -ATPase-deficient yeast. J. Biol. Chem. 274, 37270–37279. 10.1074/jbc.274.52.37270 10601292

[B45] Ramage G. Williams C. (2013). The clinical importance of fungal biofilms. Adv. Appl. Microbiol. 84, 27–83. 10.1016/B978-0-12-407673-0.00002-3 23763758

[B46] Rautemaa R. Ramage G. (2011). Oral candidosis - clinical challenges of a biofilm disease. Crit. Rev. Microbiol. 37, 328–336. 10.3109/1040841X.2011.585606 21777047

[B47] Roemer T. Krysan D. J. (2014). Antifungal drug development: challenges, unmet clinical needs, and new approaches. Cold Spring Harb. Perspect. Med. 4:a019703. 10.1101/cshperspect.a019703 24789878 PMC3996373

[B48] Rosenberg A. Ene I. V. Bibi M. Zakin S. Segal E. S. Ziv N.. (2018). Antifungal tolerance is a subpopulation effect distinct from resistance and is associated with persistent candidemia. Nat. Commun. 9:2470. 10.1038/s41467-018-04926-x 29941885 PMC6018213

[B49] Roth B. L. Poot M. Yue S. T. Millard P. J. (1997). Bacterial viability and antibiotic susceptibility testing with SYTOX green nucleic acid stain. Appl. Environ. Microbiol. 63, 2421–2431. 10.1128/AEM.63.6.2421-2431.1997 9172364 PMC168536

[B50] Schinabeck M. K. Long L. A. Hossain M. A. Chandra J. Mukherjee P. K. Mohamed S.. (2004). Rabbit model of *Candida albicans* biofilm infection: liposomal amphotericin B antifungal lock therapy. Antimicrob. Agents Chemother. 48, 1727–1732. 10.1128/AAC.48.5.1727-1732.2004 15105127 PMC400590

[B51] Schindelin J. Arganda-Carrera I. Frise E. Kaynig V. Longair M. Pietzsch T.. (2012). Fiji - an open source platform for biological image analysis. Nat. Methods 9, 676–682. 10.1038/nmeth.2019 22743772 PMC3855844

[B52] Sherry L. Kean R. McKloud E. O'Donnell L. E. Metcalfe R. Jones B. L.. (2017). Biofilms formed by isolates from patients are heterogeneous and insensitive to fluconazole. Antimicrob. Agents Chemother. 61, e01065–e01017. 10.1128/AAC.01065-17 28696240 PMC5571368

[B53] Simoes M. Pereira M. O. Vieira M. J. (2005). Action of a cationic surfactant on the activity and removal of bacterial biofilms formed under different flow regimes. Water Res. 39, 478–486. 10.1016/j.watres.2004.09.018 15644256

[B54] Struyfs C. Cools T. L. De Cremer K. Sampaio-Marques B. Ludovico P. Wasko B. M.. (2020). The antifungal plant defensin HsAFP1 induces autophagy, vacuolar dysfunction and cell cycle impairment in yeast. BBA Biomembranes 1862, 183255. 10.1016/j.bbamem.2020.183255 32145284 PMC7272304

[B55] Sud I. J. Feingold D. S. (1981). Mechanisms of action of the antimycotic imidazoles. J. Invest. Dermatol. 76, 438–441. 10.1111/1523-1747.ep12521036 7017013

[B56] Sun L. M. Cheng A. X. Wu X. Z. Zhang H. J. Lou H. X. (2009). Synergistic mechanisms of retigeric acid B and azoles against Candida albicans. J. Appl. Microbiol. 108, 341–348. 10.1111/j.1365-2672.2009.04429.x 20002912

[B57] Tits J. Cools F. De Cremer K. De Brucker K. Berman J. Verbruggen K.. (2020). Combination of miconazole and domiphen bromide is fungicidal against biofilms of resistant *Candida* spp. Antimicrob. Agents Chemother. 64, e01296–e01220. 10.1128/AAC.01296-20 32690639 PMC7508569

[B58] Tumbarello M. Fiori B. Trecarichi E. M. Posteraro P. Losito A. R. De Luca A.. (2012). Risk factors and outcomes of candidemia caused by biofilm-forming isolates in a tertiary care hospital. PLoS ONE 7:e33705. 10.1371/journal.pone.0033705 22479431 PMC3316499

[B59] Uppuluri P. Srinivasan A. Ramasubramanian A. Lopez-ribot J. L. (2011). Effects of fluconazole, amphotericin B, and caspofungin on *Candida albicans* biofilms under conditions of flow and on biofilm dispersion. Antimicrob. Agents Chemother. 55, 3591–3593. 10.1128/AAC.01701-10 21518839 PMC3122381

[B60] Vandenbosch D. Braeckmans K. Nelis H. J. Coenye T. (2010). Fungicidal activity of miconazole against *Candida* spp. biofilms. J. Antimicrob. Chemother. 65, 694–700. 10.1093/jac/dkq019 20130024

[B61] Vriens K. Cools T. L. Harvey P. J. Craik D. J. Spincemaille P. Cassiman D.. (2015). Synergistic activity of the plant defensin HsAFP1 and caspofungin against *Candida albicans* biofilms and planktonic cultures. PLoS ONE 10:e0132701. 10.1371/journal.pone.0132701 26248029 PMC4527839

[B62] Whaley S. G. Rogers P. D. (2016). Azole resistance in *Candida glabrata*. Curr. Infect. Dis. Rep. 18:41. 10.1007/s11908-016-0554-5 27761779

[B63] Yu Q. Ding X. Xu N. Cheng X. Qian K. Zhang B.. (2013). In vitro activity of verapamil alone and in combination with fluconazole or tunicamycin against *Candida albicans* biofilms. Int. J. Antimicrob. Agents 41, 179–182. 10.1016/j.ijantimicag.2012.10.009 23265915

