# Supplementary material for: Combining Miconazole and Domiphen Bromide Results in Excess of Reactive Oxygen Species and Killing of Biofilm Cells
Source: Front Cell Dev Biol. 2021 Jan 21;8:617214. doi: 10.3389/fcell.2020.617214 (PMC7858260; doi:10.3389/fcell.2020.617214)
Supplement: Supplementary file 1 [file Data_Sheet_1.PDF]

Supplementary Material

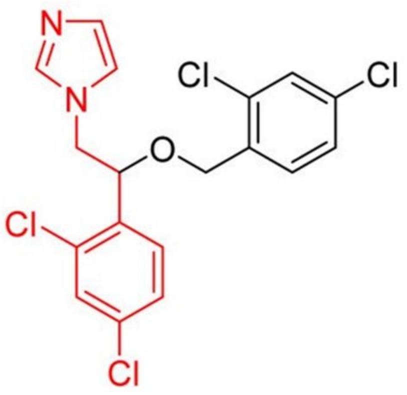

Miconazole

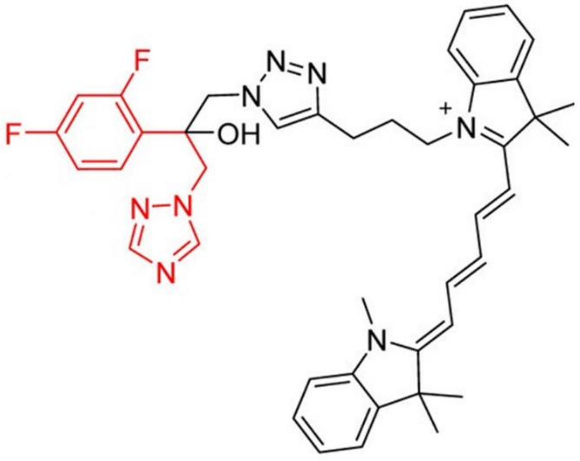

FKD

Supplementary Figure S1. Chemical structures of miconazole and fluorescently labeled imidazole derivative. The azole core structure of miconazole and fluorescently labeled imidazole derivative (FKD) are presented in red. Adapted from (Benhamou et al., 2017).

Benhamou, R. I., Bibi, M., Steinbuch, K. B., Engel, H., Levin, M., Roichman, Y., et al. (2017). Real-Time Imaging of the Azole Class of Antifungal Drugs in Live Candida Cells. *ACS Chem Biol* 12, 1769–1777. doi:10.1021/acschembio.7b00339.

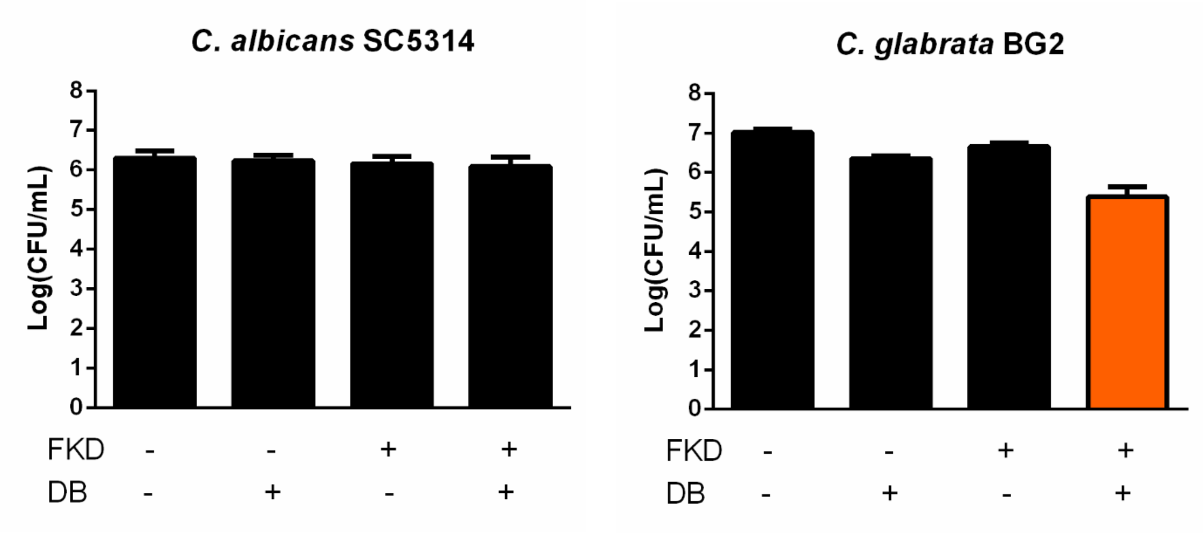

Supplementary Figure S2. Combining fluorescently labeled imidazole derivative and domiphen bromide results in fungicidal action against planktonic stationary cultures of *C. glabrata* BG2, but not against those of *C. albicans*. Planktonic stationary cultures of *C. albicans* SC5314 and *C. glabrata* BG2 were treated with 250 µM fluorescently labeled imidazole derivative (FKD) and 25 µM domiphen bromide (DB). DMSO background concentration was 2%. After 24 h treatment, the number of CFU was determined. Mean log CFU values are shown for 5 biological replicates. Statistical analysis was performed to assign significant differences upon combination treatment as compared to single compound or DMSO only treatment (‘control treatments’). A 2-way ANOVA and Tukey’s multiple comparison test was applied and significant differences ($p<0.001$) relative to the control treatments are shown in orange.

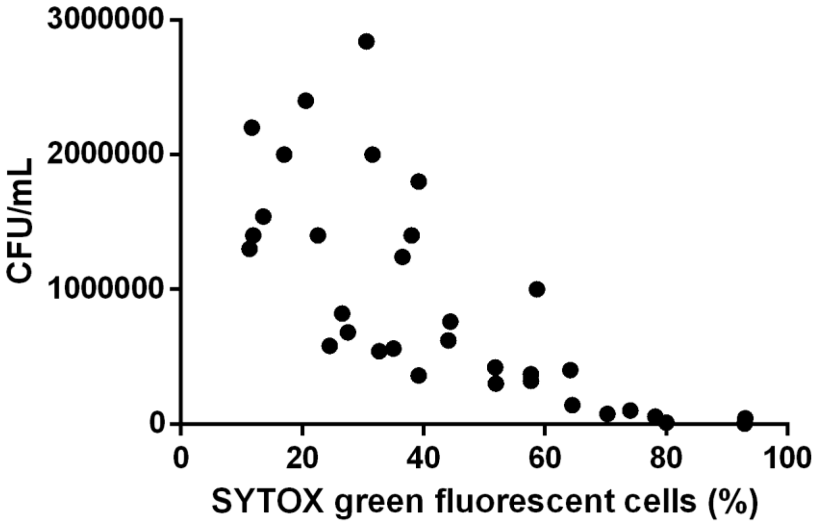

Supplementary Figure S3. Correlation between the number of CFU/mL and the amount of SYTOX green fluorescent *C. glabrata* cells upon treatment with fluorescently labeled imidazole derivative in the presence of domiphen bromide. Pearson correlation coëfficient r: -0.74; p (two-tailed) < 0.0001.

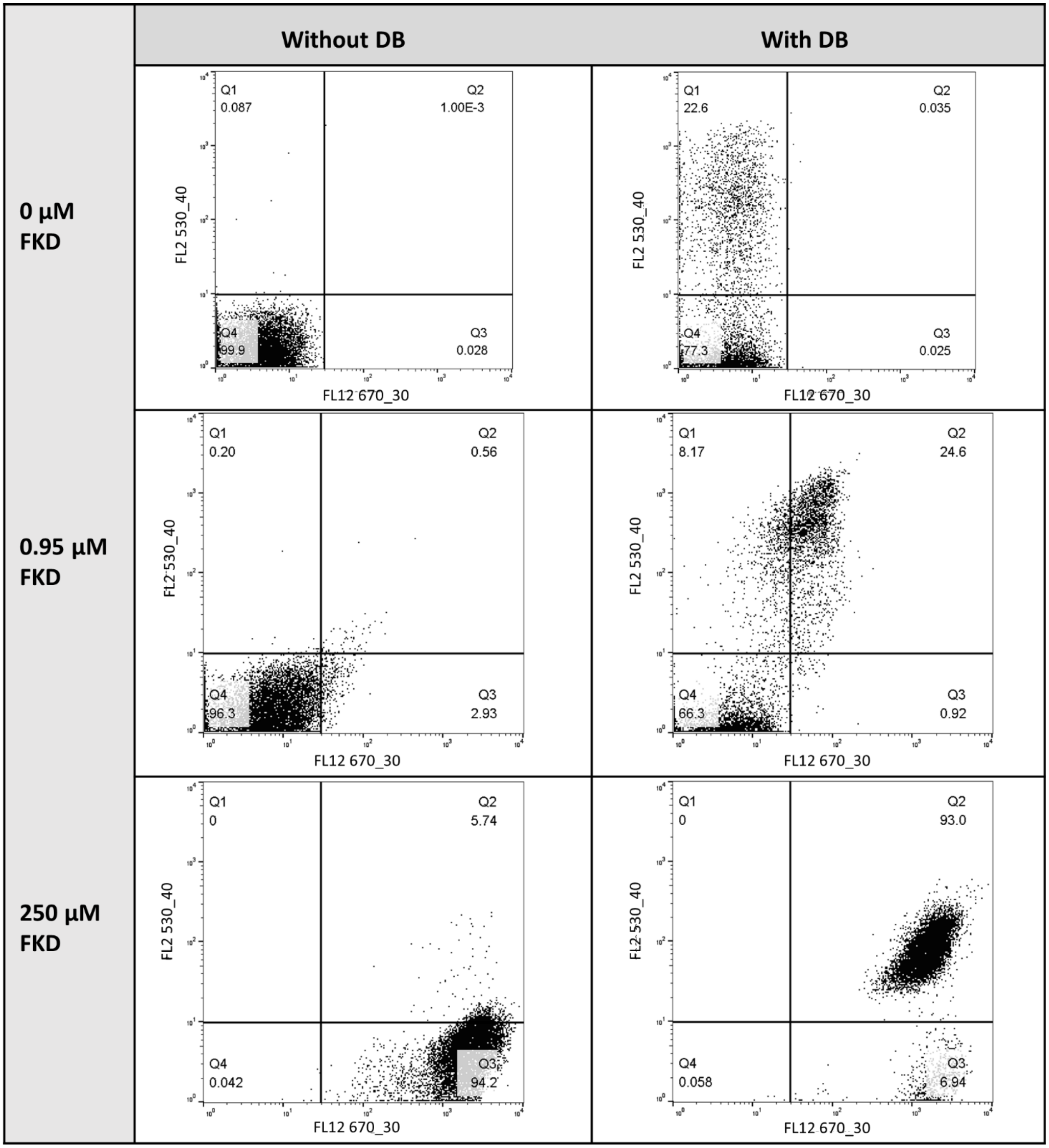

Supplementary Figure S4. Scatter plots showing the effects of DB on internalization of fluorescently labeled imidazole derivative in planktonic stationary phase *C. glabrata* BG2 cultures. *C. glabrata* BG2 planktonic cultures were treated for 2,5 h with fluorescently labeled imidazole derivative (FKD) at 0.95 µM or 250 µM in the absence or presence of 25 µM DB. DMSO background concentration was 2%. Via FACS analysis, cell cultures were divided into 4 fractions according to FKD internalization and killing, as measured by SYTOX green. Fluorescence was measured at 530/40 nm (FL2_ λex = 488 nm) and 670/30 nm (FL12_ λex = 633 nm) to detect killing (SYTOX green +) and FKD internalization (FKD +), respectively. Results are a representative of 3 biological repeats.

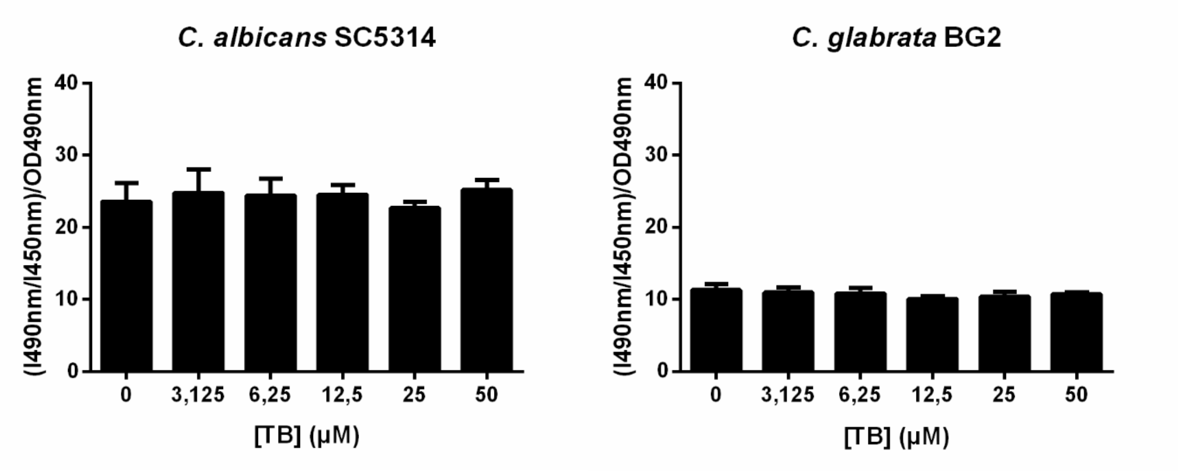

Supplementary Figure S5. Effects of TB on vacuolar integrity in *C. albicans* SC5314 and *C. glabrata* BG2 planktonic cultures in stationary phase. Stationary cultures of *C. albicans* SC5314 and *C. glabrata* BG2 were treated with a two-fold dilution series of tetraethylammonium bromide (TB) from 3.125 µM up to 50 µM for 2,5h. DMSO background concentration was 2%. The ratio of fluorescence intensity of dye BCECF,AM from excitation at 490 nm (I490nm) to fluorescence intensity from excitation at 450 nm (I450nm), both measured at an emission wavelength of 535 nm, was calculated and normalized for the cell culture biomass (OD490nm) to obtain a relative vacuolar pH value of the treated cells. Mean values +/- SEM are shown for 3 biological repeats. Statistical analysis was performed to assess a significant increase in (I490nm/I450nm)/OD490nm upon treatment with DB as compared to DMSO control treatment. A 1-way ANOVA and Dunnett's multiple comparison test was applied and no significant differences ($p<0.05$) were observed.

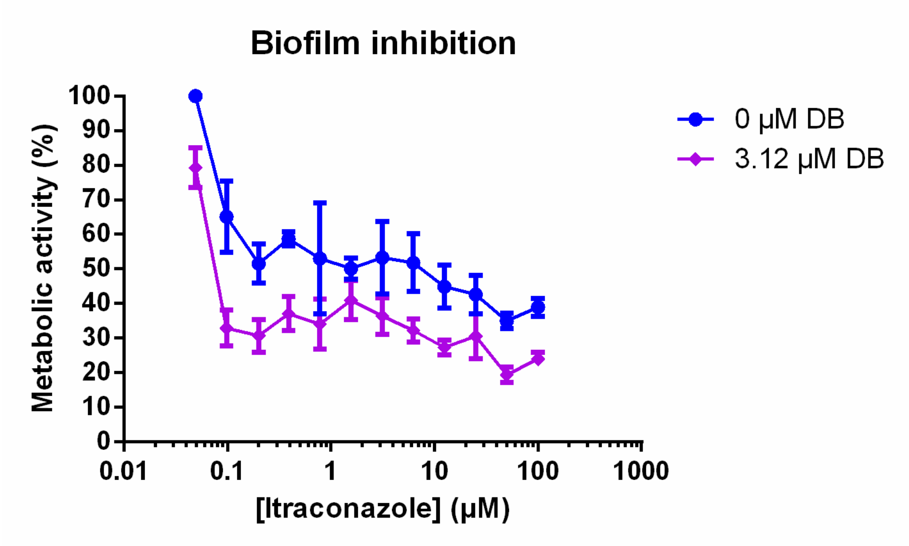

Supplementary Figure S6. Potentiation of itraconazole by DB against *C. albicans* SC5314 in a biofilm inhibition setup. *C. albicans* biofilms were grown in RPMI in the presence of itraconazole, domiphen bromide (DB) or a combination of both. Fluorescence at λ_{ex} 535 nm, λ_{em} 590 nm and sensitivity 65 were measured. Data represent metabolic activity of *C. albicans* relative to the untreated control. Mean values +/- SEM are shown for 3 biological repeats. DMSO background was 1.5%. The first data point from the left represents the percentage of metabolic activity upon itraconazole single compound treatment.
